# Automated Classification of Lung Cancer Types from Cytological Images Using Deep Convolutional Neural Networks

**DOI:** 10.1155/2017/4067832

**Published:** 2017-08-13

**Authors:** Atsushi Teramoto, Tetsuya Tsukamoto, Yuka Kiriyama, Hiroshi Fujita

**Affiliations:** ^1^School of Health Sciences, Fujita Health University, 1-98 Dengakugakubo, Kutsukake-cho, Toyoake City, Aichi 470-1192, Japan; ^2^School of Medicine, Fujita Health University, 1-98 Dengakugakubo, Kutsukake-cho, Toyoake City, Aichi 470-1192, Japan; ^3^Graduate School of Medicine, Gifu University, 1-1 Yanagido, Gifu 501-1194, Japan

## Abstract

Lung cancer is a leading cause of death worldwide. Currently, in differential diagnosis of lung cancer, accurate classification of cancer types (adenocarcinoma, squamous cell carcinoma, and small cell carcinoma) is required. However, improving the accuracy and stability of diagnosis is challenging. In this study, we developed an automated classification scheme for lung cancers presented in microscopic images using a deep convolutional neural network (DCNN), which is a major deep learning technique. The DCNN used for classification consists of three convolutional layers, three pooling layers, and two fully connected layers. In evaluation experiments conducted, the DCNN was trained using our original database with a graphics processing unit. Microscopic images were first cropped and resampled to obtain images with resolution of 256 × 256 pixels and, to prevent overfitting, collected images were augmented via rotation, flipping, and filtering. The probabilities of three types of cancers were estimated using the developed scheme and its classification accuracy was evaluated using threefold cross validation. In the results obtained, approximately 71% of the images were classified correctly, which is on par with the accuracy of cytotechnologists and pathologists. Thus, the developed scheme is useful for classification of lung cancers from microscopic images.

## 1. Introduction 

Lung cancer is a leading cause of death for both males and females worldwide [1]. Primary lung cancers are divided into two major types: small cell lung cancer and non-small cell lung cancer. Recent improvements in chemotherapy and radiation therapy [2] have resulted in the latter being further classified into adenocarcinoma, squamous cell carcinoma, and large cell carcinoma [3]. It is often difficult to precisely differentiate adenocarcinoma and squamous cell carcinoma in terms of their morphological characteristics, which requires immunohistochemical evaluation. Cytodiagnosis is advantageous for cytological evaluation of small cell carcinoma compared to histological specimen, often showing crushed small cell cancer cells. For definitive and precise diagnosis, cooperation of cytological evaluation and histopathological diagnosis—which are independent techniques—is indispensable. There are many varieties of morphologies among these cancer cells. Computer-aided diagnosis (CAD) can be a useful tool for avoiding misclassification. Among the four major types of carcinoma, large cell carcinoma is the easiest to detect because of its severe atypism. We therefore concentrate on classification of the other three types—adenocarcinoma, squamous cell carcinoma, and small cell carcinoma—which are sometimes confused with each other in the cytological specimen.

CAD provides a computerized output as a “second opinion” to support a pathologist's diagnosis and helps clinical technologists and pathologists to evaluate malignancies accurately. In this study, we focused on automated classification of cancer types using microscopic images for cytology.

Various studies that apply CAD methods to pathological images have been conducted [4–7]. Barker et al. [5] proposed an automated classification method for brain tumors in whole-slide digital pathology images. Ojansivu et al. [6] investigated automated classification of breast cancer from histopathological images. Ficsor et al. [7] developed a method for automated classification of inflammation in colon histological sections based on digital microscopy. However, to the best of our knowledge, no method has been developed to classify lung cancer types from cytological images.

Deep learning is well known to give better performance than conventional image classification techniques [8, 9]. For example, Krizhevsky et al. [8] won the 2012 ImageNet Large-Scale Visual Recognition Challenge (ILSVRC) using a deep convolutional neural network (DCNN) to classify high-resolution images. In addition, many research groups have investigated the application of DCNNs to medical images [10–13].

Various CAD methods have been proposed for pathological images using deep learning techniques. For example, Ciresan et al. developed a system that uses convolutional neural networks for mitosis counting in primary breast cancer grading [14]. Wang et al. combined handcrafted features and deep convolutional neural networks for mitosis detection [15]. Ertosun and Rubin proposed an automated system for grading gliomas using deep learning [16]. Xu et al. developed a deep convolutional neural network that segments and classifies epithelial and stromal regions in histopathological images [17]. Litjens et al. investigated the effect of deep learning for histopathological examination and verified that its performance was excellent in prostate cancer identification and breast cancer metastasis detection [18].

To our knowledge, DCNNs have not been applied to cytological images for lung cancer classification. In this study, we developed an automated classification scheme for lung cancers in microscopic images using a DCNN.

## 2. Materials and Method

### 2.1. Image Dataset

Seventy-six (76) cases of cancer cells were collected by exfoliative or interventional cytology under bronchoscopy or CT-guided fine needle aspiration cytology. They consisted of 40 cases of adenocarcinoma, 20 cases of squamous cell carcinoma, and 16 cases of small cell carcinoma. Final diagnosis was made in all cases via a combination of histopathological and immunohistochemical diagnosis. Specifically, biopsy tissues, simultaneously collected with cytology specimen, were fixed in 10% formalin, dehydrated, and embedded in paraffin. The 3 *μ*m tissue sections were subjected to immunohistochemical analysis for some cases. Cancer lesions were judged as adenocarcinoma if TTF-1 and/or napsin A were positive and diagnosed as squamous cell carcinoma if p40 and/or cytokeratin 5/6 were present. Positivity of neuroendocrine markers including chromogranin A, synaptophysin, and CD56 was suggestive of small cell carcinoma.

The cytological specimens were prepared with a liquid-based cytology (LBC) system using BD SurePath liquid-based Pap Test (Beckton Dickinson, Durham, NC, USA), and were stained using the Papanicolaou method. Using a digital still camera (DP70, Olympus, Tokyo, Japan) attached to a microscope (BX51, Olympus) with ×40 objective lens, 82 images of adenocarcinoma, 125 images of squamous cell carcinoma, and 91 images of small cell carcinoma were collected in JPEG format. The initial matrix size of each JPEG image was 2040 × 1536 pixels.

Subsequently, 768 × 768 pixels square images were generated by cropping and were further resized to 256 × 256 pixels. Subsequently, duplicate 768 × 768 pixels square images were cut from the original image in order not to cause overlap therefrom. Finally, they were resized to 256 × 256 pixels.

This study was approved by an institutional review board, and patient agreements were obtained under the condition that all data were anonymized (number HM16-155).

### 2.2. Data Augmentation

Training of a DCNN requires that a sufficient amount of training data be available. If only a small amount of training data is used, overfitting may result. To prevent overfitting owing to the limited number of images, training data were augmented by image manipulation [8, 13]. The images obtained by microscope are direction-invariant and the sharpness of the target cell in each image varies according to the position of the focal plane of the microscope. Therefore, we performed data augmentation by rotating, inverting, and filtering the original image.

The rotation pitch for the image was determined such that the number of augmented images was the same for the three cancer classes. In addition, the images were flipped, resulting in the final number of images being twice that of the original. For filtering, Gaussian filter (standard deviation of Gaussian kernel = 3 pixels) and a convolutional edge enhancement filter with center weight 5.4 and the 8-surrounding weight of −0.55 were applied to the images.

### 2.3. Network Architecture

**Figure 1:**
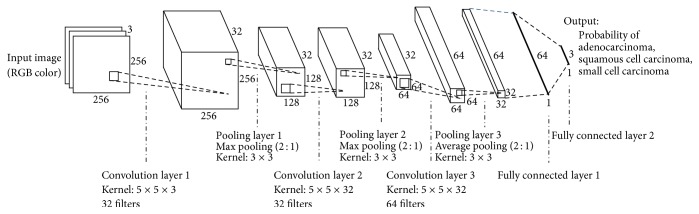
Architecture of the deep convolutional neural network used for cancer-type classification.

The architecture of the DCNN used for cancer-type classification is shown in Figure 1. It consists of three convolution layers, three pooling layers, and two fully connected layers. Color microscopic images are given to the input layer of the DCNN. The filter size, number of filters, and stride for each layer are specified in Figure 1. For example, convolution layer 1 uses 32 filters with a 5 × 5 × 3 kernel, resulting in a feature map of 256 × 256 × 32 pixels; pooling layer 1 conducts subsampling (resampling) that outputs the maximum value in a 3 × 3 kernel for every two pixels, reducing the matrix size of the feature map to 128 × 128 × 32 pixels. Each convolution layer is followed by a rectified linear unit (ReLU). After three convolution layers and three pooling layers, there are two fully connected layers consisting of a multilayer perceptron. In the last layer, the probabilities of cancer types (adenocarcinoma, squamous cell carcinoma, and small cell carcinoma) are obtained using a softmax function. In the training, we employed the dropout method (dropout rate = 50% for full connection layers) to prevent overfitting.

The DCNN was trained using the dedicated training program bundled in the Caffe package [19] on Ubuntu 16.04 and accelerated by a graphic processing unit (NVIDIA GeForce GTX TITAN X with 12 GB of memory). The number of epochs and training time were 60,000 and 8 hours, respectively.

## 3. Results

For classification of the three cancer types, DCNN was trained and evaluated using augmented data and original data, respectively. Its classification performance was evaluated via threefold cross validation. In this process, 298 images were randomly divided into three groups. However, images taken from the same specimen belonged to the same group.

The original number of images in each dataset is listed in Table 1. By data augmentation, the number of images for each class was unified to approximately 5000.

**Figure 2:**
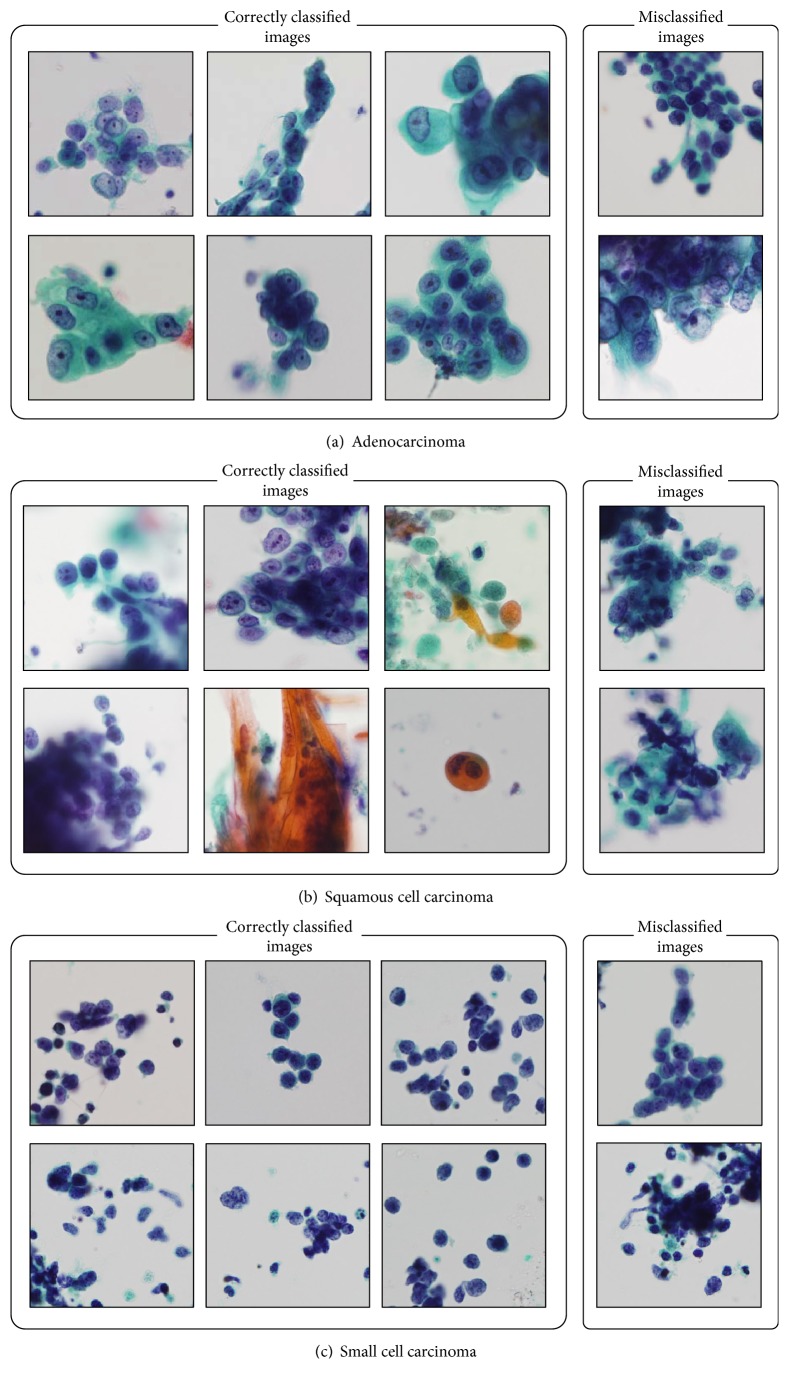
Sample images of correctly classified and misclassified carcinoma.

Figure 2 shows sample images of correctly classified and misclassified cancer types obtained using the data augmentation method. The classification confusion matrix is shown in Table 2. It can be seen that squamous cell cancer was often mistaken for adenocarcinoma.

Table 3 shows the classification accuracy for the original and the augmented images, respectively. In the results obtained using the augmented images, the classification accuracies of adenocarcinoma, squamous cell carcinoma, and small cell carcinoma were 89.0%, 60.0%, and 70.3%, respectively; the total correct rate was 71.1%. Furthermore, applying augmentation (rotation, flipping, and filtering) provided better classification results.

## 4. Discussion

Using the DCNN, 70% of lung cancer cells were classified correctly. Most of the correctly classified images have typical cell morphology and arrangement. In traditional cytology, pathologists perform classification of small cell carcinoma and non-small cell carcinoma. The classification accuracy rate in this case is 255/298 = 85.6%, which is considered to be sufficient. Of the three types of lung cancers, classification accuracy was highest for adenocarcinoma and lowest for squamous cell carcinoma. This result may be related to the variation of images used for training. The number of images of squamous cell carcinoma was less than that for adenocarcinoma and data augmentation improved classification performance by 15%. We plan to increase the number of cases of adenocarcinoma and squamous cell carcinoma used in future studies. For small cell carcinoma, classification accuracy was highest in the results without data augmentation; the accuracy decreased marginally with augmentation. It has distinctive features in that small cell carcinoma has only little cytoplasm and a small nucleus. Therefore, its image characteristics could be understood by the DCNN from even a small number of images.

The classification accuracy of 71.1% is comparable to that of a cytotechnologist or pathologist [20, 21]. It is noteworthy that DCNN is able to understand cell morphology and placement of cancer cells solely from images without prior knowledge and experience of biology and pathology. The methodology using CNN has already been established. However, to the best of our knowledge, no work has been carried out on automated classification using cytological images. Our experimental results indicate that our overall accuracy rate is more than 70%. This is a satisfactory result because cytological diagnosis of lung cancer is a difficult task for pathologists. Therefore, our method will be useful in assisting with cytological examination in lung cancer diagnosis.

In future studies, we plan to analyze the image features that DCNN focuses on during classification in order to reveal the classification mechanism in detail. We classified lung cancer types in whole images with multiple cells. However, it is also possible to perform feature analysis and classification by focusing on individual cells. Therefore, we hope to develop a method to comprehensively classify cells and arrays of cells.

## 5. Conclusion

In this study, we developed an automated classification scheme for lung cancers in microscopic images using a DCNN. Evaluation results showed that approximately 70% of images were classified correctly. These results indicate that DCNN is useful for classification of lung cancer in cytodiagnosis.

## Figures and Tables

**Table 1:** Number of images in each dataset for cross validation.

	Set 1		Set 2		Set 3	
	Original	Augmented	Original	Augmented	Original	Augmented
Adenocarcinoma	28	5280	28	5184	26	5040
Squamous cell carcinoma	42	5478	37	5220	46	5310
Small cell carcinoma	26	5070	33	5280	32	5214

**Table 2:** Confusion matrix of classification results.

	Adenocarcinoma	Squamous cell carcinoma	Small cell carcinoma
Adenocarcinoma	73 (89.0%)	8 (9.8%)	1 (1.2%)
Squamous cell carcinoma	35 (28.0%)	75 (60.0%)	15 (12.0%)
Small cell carcinoma	7 (7.7%)	20 (22.0%)	64 (70.3%)

**Table 3:** Classification accuracies trained by original and augmented images.

	Classification accuracy [%]	
	Original	Augmented
Adenocarcinoma	73.2	89.0
Squamous cell carcinoma	44.8	60.0
Small cell carcinoma	75.8	70.3
Total	62.1	71.1
